# Membrane Proteins Are Dramatically Less Conserved than Water-Soluble Proteins across the Tree of Life

**DOI:** 10.1093/molbev/msw164

**Published:** 2016-08-08

**Authors:** Victor Sojo, Christophe Dessimoz, Andrew Pomiankowski, Nick Lane

**Affiliations:** ^1^CoMPLEX, University College London, London, United Kingdom; ^2^Department of Genetics, Evolution and Environment, University College London, London, United Kingdom; ^3^Systems Biophysics, Faculty of Physics, Ludwig-Maximilian University of Munich, Munich, Germany; ^4^Department of Ecology and Evolution, University of Lausanne, Lausanne, Switzerland; ^5^Center for Integrative Genomics, University of Lausanne, Lausanne, Switzerland

**Keywords:** membrane proteins, orthologs, homeostasis, evolution, adaptation

## Abstract

Membrane proteins are crucial in transport, signaling, bioenergetics, catalysis, and as drug targets. Here, we show that membrane proteins have dramatically fewer detectable orthologs than water-soluble proteins, less than half in most species analyzed. This sparse distribution could reflect rapid divergence or gene loss. We find that both mechanisms operate. First, membrane proteins evolve faster than water-soluble proteins, particularly in their exterior-facing portions. Second, we demonstrate that predicted ancestral membrane proteins are preferentially lost compared with water-soluble proteins in closely related species of archaea and bacteria. These patterns are consistent across the whole tree of life, and in each of the three domains of archaea, bacteria, and eukaryotes. Our findings point to a fundamental evolutionary principle: membrane proteins evolve faster due to stronger adaptive selection in changing environments, whereas cytosolic proteins are under more stringent purifying selection in the homeostatic interior of the cell. This effect should be strongest in prokaryotes, weaker in unicellular eukaryotes (with intracellular membranes), and weakest in multicellular eukaryotes (with extracellular homeostasis). We demonstrate that this is indeed the case. Similarly, we show that extracellular water-soluble proteins exhibit an even stronger pattern of low homology than membrane proteins. These striking differences in conservation of membrane proteins versus water-soluble proteins have important implications for evolution and medicine.

## Introduction

Biological membranes form the boundary between the cell and its surroundings, and their embedded proteins constitute an active link to the environment, with crucial roles in reproduction, bioenergetics, transport, signaling, and catalysis (Mitchell 1957, 1961; Singer and Nicolson 1972; Hedin et al. 2011). Over half of all known drug targets are membrane proteins (Overington et al. 2006). Their study is therefore central to our understanding of the origins and evolution of life, as well as to physiology and medicine.

Previous studies have shown that the subcellular localization of a protein is a strong predictor of its evolutionary rate. Extracellular proteins secreted from the cell evolve faster than intracellular proteins in both mammals and yeast, as do the external parts of membrane proteins (Tourasse and Li 2000; Julenius and Pedersen 2006; Liao et al. 2010), but the reasons are unclear. Structural and packing constraints undoubtedly play a role, with the exposure of amino-acid residues to the solvent (Oberai et al. 2009; Franzosa et al. 2013), as well as the sub-cellular localization of the proteins and their portions (Julenius and Pedersen 2006; Liao et al. 2010) being the strongest predictors of evolutionary rate. Membrane proteins also diverge faster than intracellular water-soluble proteins in parasites, where surface interactions evolve under pressure to avoid detection by the host (Volkman et al. 2002; Plotkin et al. 2004). This pattern may be specific to the “red-queen” dynamics of parasitic interactions, that is, the need for constant adaptation merely to maintain fitness. Taken together, however, these disparate findings suggest that evolution might generally occur faster outside the cell, and hint at the operation of a wider evolutionary mechanism.

**Fig. 1:**
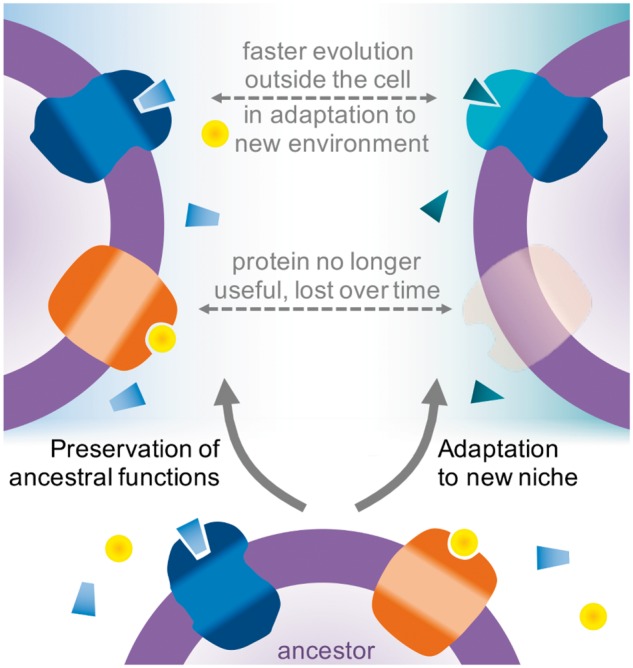
Two-fold effect of adaptation causes faster evolution of external sections and loss of homology in membrane proteins. Adaptation to new functions and niches causes faster evolution for outside-facing sections (top), potentially contributing to divergence beyond recognition. Other proteins may provide no advantage in the new environment, and could be lost entirely over time (center). For simplicity, the species on the left is assumed to remain functionally identical to the common ancestor (bottom).

Here, we test the hypothesis that protein evolution is faster outside the cell as a result of adaptation to changing environments (fig. 1, top). Over evolutionary time, the interior of the cell remains stable compared with the exterior, which is forced to change in response to shifting biogeochemical processes, migration with colonization of new niches, and other biotic interactions. This leads to the faster evolution of secreted water-soluble proteins and outside-facing sections of membrane proteins. The utility of a protein will also depend on the specific environment, potentially leading to greater loss of membrane-bound proteins over time as environments change (fig. 1, middle). We have analyzed large data sets of orthologs to evaluate the conservation of membrane proteins relative to water-soluble proteins across the entire tree of life, to test whether faster evolution outside the cell is driven by adaptation to new environments and functions.

## Results

### Membrane Proteins Are Shared by Fewer Species in All Three Domains of Life

**Fig. 2:**
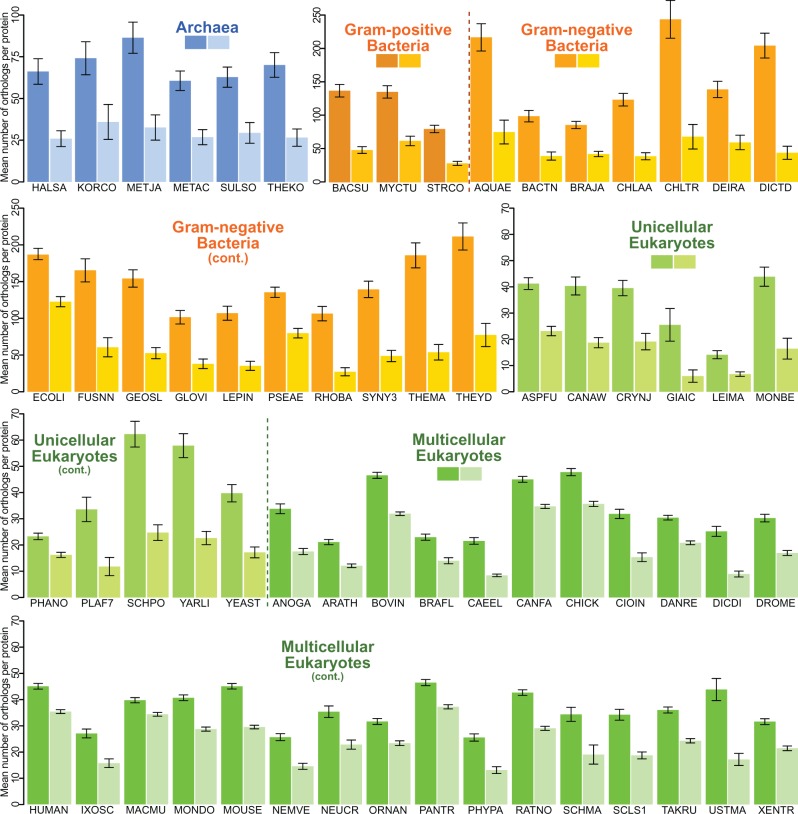
Membrane proteins have fewer orthologs in all three domains of life. The mean size of OMA Ortholog Groups (OGs) is substantially smaller for membrane proteins in all 64 species in the EMBL-EBI’s list of reference proteomes studied (2 of the 66 species were not found in OMA at the time of this analysis). Five-letter codes are OMA species identifiers; details in supplementary table S1, Supplementary Material online. Dark shade: water-soluble (WS); light shade: membrane proteins (MP). Data represented as the mean number of orthologs that WSs and MPs of each genome have in OMA±2×SEM (standard error of the mean).

To study the evolution of membrane proteins across the tree of life, we downloaded the 883,176 pre-computed ortholog groups (OGs) for all 1,706 species from the three domains of life present in the OMA database (Altenhoff et al. 2015). We separately obtained the full list of 66 species in the EMBL-EBI list of reference proteomes (www.ebi.ac.uk/reference_proteomes), and extracted the OMA OGs for each protein of each species, where present (supplementary table S1, Supplementary Material online). We classified each protein sequence as either a membrane protein (MP) or a water-soluble protein (WS) using the predictions of the TMHMM algorithm (Krogh et al. 2001). We then determined the number of orthologs found for each protein (i.e., the size of the ortholog cluster, or OG, for each protein) independently for each species. We find that, in all cases of all three domains of life (archaea, Gram-positive and Gram-negative bacteria, as well as unicellular and multicellular eukaryotes), the mean number of orthologs is substantially smaller for MPs than for WSs (fig. 2 and supplementary table S1, Supplementary Material online); that is, membrane proteins are shared by fewer species on average (paired *t*-test: *t *= 8.05; d*f* = 63; *P* = 2.88·10^−11^; *r* = 0.712). A simple protein–protein BLAST (BLASTp) search (Altschul et al. 1990) against the full nonredundant (nr) NCBI protein database confirms these findings (supplementary fig. S1, Supplementary Material online).

**Fig. 3:**
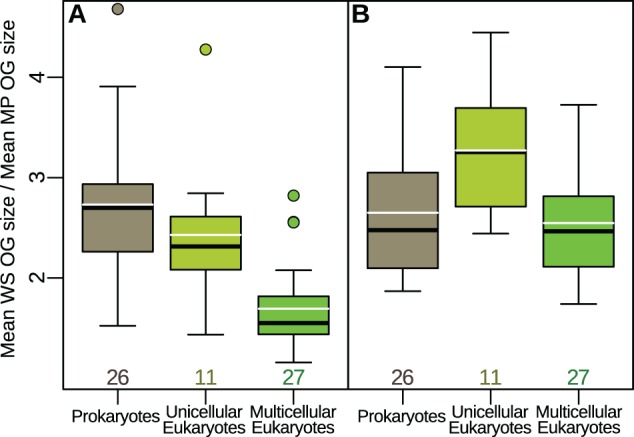
Water-soluble orthologous groups are substantially larger on average than membrane-protein groups, but the effect decreases as organismal complexity increases. Dividing the average size of water-soluble orthologous groups (OGs) of each species over the corresponding average size of membrane-protein OGs gives an indication of the magnitude of the effect in figure 2 for the different groups of species. (*A*) The ratio of the mean sizes of water-soluble over membrane protein OGs is > 1 for all species studied (i.e., each WS bar is always larger than its corresponding MP bar in figure 2), but the effect decreases as cellular and organismal complexity increase, from prokaryotes to unicellular eukaryotes, to multicellular eukaryotes. (*B*) Filtering for orthologous groups composed of both eukaryotes and prokaryotes keeps the relationship between unicellular and multicellular eukaryotes and indeed increases the effect, whereas prokaryotes remain largely unaltered. This suggests that membrane proteins ancestral to eukaryotes (i.e., with ancestors in archaea or bacteria) have been lost more often than their water-soluble counterparts. Bold black lines represent the median, white lines the mean, and boxes and whiskers are standard in R at a ± 1.5*IQR (inter-quartile range) threshold. Numbers below the boxes indicate sample sizes.

This dramatic reduction in the conservation of membrane proteins is widespread across the entire tree of life, but the effect decreases as cellular or organismal complexity increases. Water-soluble proteins have on average 2.7 times more orthologs than membrane proteins in prokaryotes. The factor decreases to 2.4 in unicellular eukaryotes, and to 1.7 in multicellular eukaryotes (fig. 3*A*; one-way analysis of variance: F(2,61)= 21.07; *P* = 1.1·10^−7^; ω^2 ^=^ ^0.149). Filtering for proteins shared by eukaryotes and at least one of the prokaryotic domains produces the results in figure 3*B*. While prokaryotes are largely unaltered and the difference between unicellular and multicellular eukaryotes remains, the effect becomes larger overall for eukaryotes. That is, potentially ancestral proteins in eukaryotes (namely with orthologs in either archaea or bacteria) are more likely to be lost if they are membrane-bound.

**Fig. 4:**
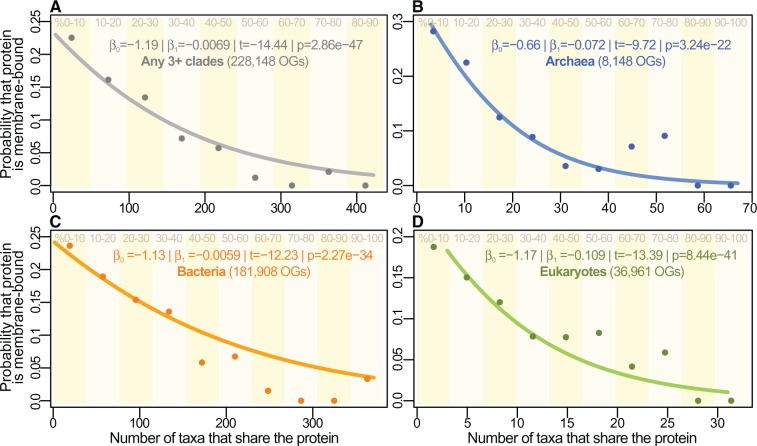
The probability of a protein being membrane-bound falls with wider distribution. (*A*) A logistic regression shows that the probability that a gene is a membrane protein falls significantly with increasing number of clades sharing it, for OGs shared by any 3 or more separate clades. The pattern remains when considering each of the three domains separately (*B–D*). The points and vertical stripes correspond to the proportions of MPs amongst genes shared by increasingly large numbers of clades, divided in 10% bins. No proteins retrieved were shared by over 90% of the 489 taxa in (*A*). In all cases, the final bins have proportion zero, i.e., no highly shared proteins are membrane-bound. Note that the points and bins are provided for reference only: logistic regressions were performed on the individual ortholog clusters (i.e., the probability curves were derived independently, see Materials and Methods section).

We performed a logistic regression on the entire pre-computed OMA ortholog data set to estimate the probability that any given protein is membrane-bound as the number of clades sharing it increases. We find that the more universal the protein, the less likely it is to be membrane-bound (fig. 4).

Since ortholog discovery depends on the successful detection of homologs using tools such as BLAST, the lower homology of membrane proteins we report could have two main causes (fig. 1). First, it is possible that membrane proteins evolve faster and hence their more divergent sequences are picked up less frequently by homology-identification algorithms. Second, some of the absences may be true gene losses, such that the orthologs are not found because they are genuinely no longer there. We show that both mechanisms are at play.

### Faster Evolution of Membrane Proteins and Their Outside-Facing Sections

**Fig. 5:**
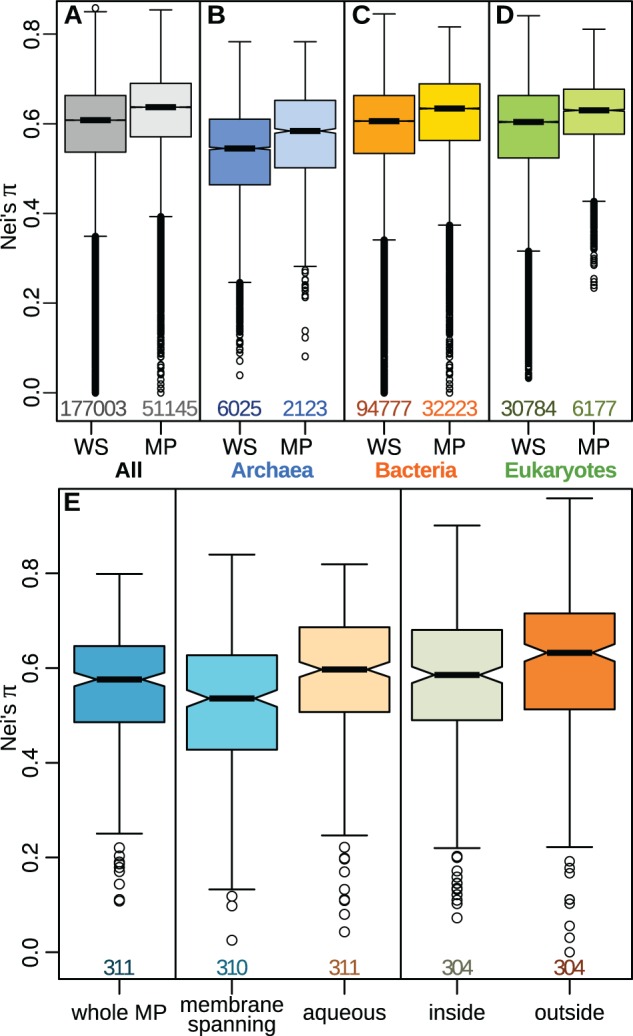
Membrane proteins evolve faster, especially in their external sections. (*A–D*) Nei’s sequence diversity measure (π) is higher for membrane proteins (MP) than for water-soluble proteins (WS) in the full set of OMA OGs (*A*) as well as for each of the three domains of life separately (*B–D*), indicating that evolution occurs faster for MPs. (*E*) For sections of membrane-protein OMA OGs annotated from the structures in the PDBTM database, Nei’s π shows that aqueous sections evolve faster overall than membrane-spanning sections. Splitting the aqueous sequences into outside- and inside-facing sections confirms that regions exposed to the environment evolve faster than those facing the cytosol. Boxplot ranges as in figure 3 with notches at the 95% confidence-interval around the median. All comparisons of WS to MP in (*A–D*), as well as inside and outside portions to each other or to membrane-spanning portions in (*E*) had *P*<0.001. Digits below the boxes indicate the numbers of orthologous groups.

To investigate whether the patterns above are due to membrane proteins having a higher divergence rate overall, we calculated Nei’s sequence-diversity measure (π, Nei and Li 1979) for the 228,148 OMA OGs shared by any three or more species. The results confirm previous reports on data sets with more limited phylogenetic ranges (Volkman et al. 2002; Julenius and Pedersen 2006) that membrane proteins diverge more quickly than water-soluble proteins (Welch’s *t*-test: *t* = 14.08, d*f* = 14261.09; *P* = 2.59·10^−45^; *r* = 0.12, fig. 5*A*); this result is consistent across the three domains of life (fig. 5*B*–*D*).

While the TMHMM algorithm has been shown to infer trans-membrane helical (TMH) regions with very high accuracy (Krogh et al. 2001), discerning the inside- versus outside-facing aqueous regions of TMH proteins is substantially more challenging. We downloaded the full nonredundant set of sequences and annotations from the trans-membrane protein data bank (PDBTM, pdbtm.enzim.hu) (Tusnády et al. 2004), to assess the evolution of the three main regions of trans-membrane proteins: inside-facing aqueous, membrane-spanning, and outside-facing aqueous. Briefly, this database has annotations, where available, for the sub-cellular localization of each amino acid in all membrane-protein structures deposited in the Protein Data Bank (PDB, www.rcsb.org) (Berman et al. 2000; Rose et al. 2015). We performed a BLASTp search of the sequence of each PDB structure against our subset of the OMA database, aligned the sequences of the best-matching orthologous groups, and sliced the alignments vertically to obtain the inside-facing, membrane-spanning, and outside-facing regions, plus an “aqueous” assemblage constructed by concatenating the inside and outside portions (see Materials and Methods section for details). We next calculated Nei’s sequence-diversity measure (π) for each section of the protein alignments (fig. 5*E*). The results confirm that aqueous regions evolve faster than membrane-spanning regions (paired *t*-test: *t* = 8.87; d*f* = 309; *P* = 5.95·10^−17^; *r* = 0.450). Amongst the aqueous regions, both of which have faster rates than the membrane-spanning regions overall, the outside-facing portions evolve faster than their inside-facing counterparts (fig. 5*E*; paired *t*-test: *t* = 3.76; d*f* = 296; *P* = 2.07·10^−4^; *r* = 0.213). These results are confirmed using an additional estimate computed by building trees for the sliced alignment portions and averaging the branch lengths of all nodes within each tree (supplementary fig. S2, Supplementary Material online; see Materials and Methods section for details). As before, aqueous regions are shown to evolve faster than membrane-spanning regions (paired *t*-test: *t* = 10.2109; d*f* = 371; *P* = 1.40·10^−16^; *r* = 0.411), whereas specifically the outside-facing sections once more have faster rates than their corresponding inside-facing sections (paired *t*-test: *t* = 4.63; d*f* = 359; *P* = 5.22·10^−6^; *r* = 0.237).

To control for potential errors in the automatic annotations of PDBTM, we repeated our analysis by manually annotating the 3 main regions (inside, outside, and membrane-spanning) of 12 membrane proteins that are highly shared in OMA, including 1 outer-membrane beta-barrel porin and 11 trans-membrane helical proteins. The closest-matching structural file was found by BLASTp search against the PDB subset on the NCBI server. The subcellular location of each amino-acid residue was then assigned by inspecting the PDB structures against the information in the corresponding primary literature (supplementary table S2, Supplementary Material online). Orthologs were assigned from the corresponding OMA OG (except in the case of OmpF, whose homologs were obtained from a BLASTp search against the nr database). The homologous sequences were aligned to the PDB sequence, alignments sliced and evolutionary rates estimated using Nei’s π. In 10 of the 12 proteins hand-annotated in this way, evolution occurs faster for outside-facing than for inside-facing aqueous regions (supplementary fig. S3, Supplementary Material online; paired *t*-test: *t* = 4.97; d*f* = 11; *P* = 4.25·10^−4^; *r* = 0.832). Using the mean branch lengths of trees as an alternative estimate of evolutionary rates, all 12 proteins show faster rates in the outside-facing regions than in their inside-facing counterparts (supplementary fig. S4, Supplementary Material online; paired *t*-test: *t* = 4.71; d*f* = 11; *P* = 6.37·10^−4^; *r* = 0.818).

These findings are again widespread across the tree of life, and apply to multiple types of proteins. We note that these patterns hold true despite the fact that some aqueous proteins are exported from the cell and predictably evolve faster (Julenius and Pedersen 2006), whereas some membrane proteins, especially in eukaryotes, sit on organellar membranes (hence presumably evolve slower).

### Extracellular Water-Soluble Proteins Have Fewer Orthologs than Membrane Proteins

**Fig. 6:**
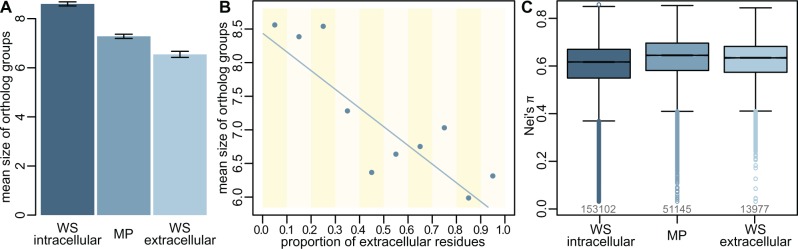
Extracellular proteins evolve faster and are shared by fewer species on average. (*A*) The mean ortholog cluster size is smaller for extracellular water-soluble than for membrane-bound proteins, whereas intracellular (cytosolic) proteins are shared by more species on average. (*B*) Binning proteins by the proportion of their amino-acid residues that are extracellular produces the mean OG sizes represented by the points, whereas the line is a simple linear regression on these points. See Materials and Methods section for details. (*C*) The evolutionary rates, estimated by Nei’s π, are higher for exported water-soluble proteins than for their intracellular counterparts, whereas membrane proteins show a slightly higher rate overall. Digits below the boxes indicate the numbers of orthologous groups.

To estimate the effect of extracellularity, we used the predictions of the SignalP package (Petersen et al. 2011). Briefly, this software detects fragments of amino-acid sequences likely to target proteins to the secretory pathway. SignalP detects these signal peptides in most Gram-positive and Gram-negative bacteria, as well as eukaryotes (note that the software is presently unable to reliably predict signal peptides in archaea). We re-classified all water-soluble OMA OGs as either intracellular (i.e., cytosolic), or extracellular (i.e., containing a signal peptide and not being a membrane protein), based on SignalP predictions. Extracellular proteins are shared on average by notably fewer clades than intracellular (most simply cytosolic) proteins (fig. 6*A*; mean OG size of intracellular WS proteins 8.60 versus 6.55 for extracellular; Welch’s *t*-test: *t* = 27.62, d*f* = 29602.0; *P* = 7.40·10^−166^; *r* = 0.16). Membrane proteins are intermediate: less widespread than intracellular proteins, but more so than extracellular ones. Similarly, grouping proteins by the proportion of their residues that are exposed to the environment produces a pattern of falling phylogenetic spread as extracellular exposure increases (fig. 6*B*; linear regression on data binned as described in Materials and Methods; F(1,8)=25.45; *P* = 0.00147; *r* = 0.859). The evolutionary rates, measured by Nei’s π, are faster for extracellular than for intracellular water-soluble proteins (fig. 6*C*; mean for intracellular WS proteins 0.592 versus 0.610 for extracellular; Welch’s *t*-test: *t*=22.67, d*f* = 17163.09; *P* = 3.70·10^−112^; *r* = 0.171), whereas membrane proteins show a slightly higher rate.

### Membrane Proteins Have Been Lost More Often within Closely Related Species

The results in figure 5 suggest that the higher evolutionary rates of membrane proteins could, through divergence beyond recognition, lead to the loss of homology reported earlier (fig. 2 and supplementary fig. S1, Supplementary Material online). To determine whether true gene loss has occurred as well, we repeated the presence–absence analysis (fig. 2) on sets of proteins predicted to be ancestral to closely related species and strains. We selected all prokaryotic clades with 10 or more closely related species in OMA, and assumed that proteins shared by more than half of the members of the clade were ancestral (see Materials and Methods section). We considered that any clades that do not share such ancestral proteins represent true gene losses, on the assumption that in closely related strains and species orthologs are unlikely to have diverged beyond recognition.

**Fig. 7:**
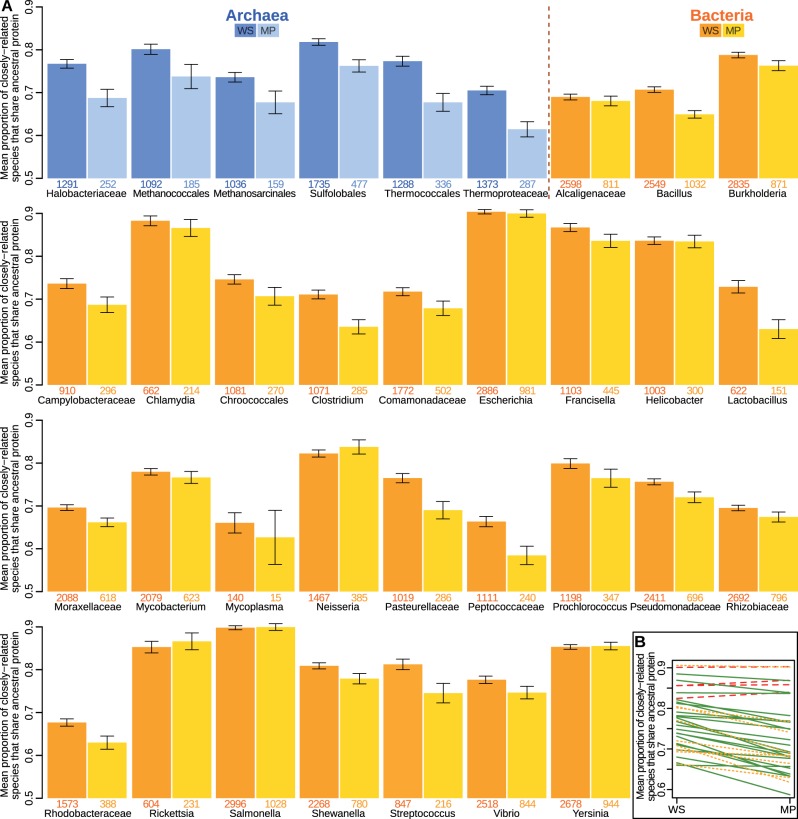
Ancestral membrane proteins have been lost more frequently. (*A*) Predicted ancestral proteins (defined as shared by at least half of the members of a clade), are shared by a smaller proportion of members in the clade if they are membrane proteins, for 31 of the 35 groups studied (exceptions are *Neisseria*, *Rickettsia*, *Salmonella*, and *Yersinia*). Dark shade: water-soluble; light shade: membrane proteins. First six pairs (blue) are archaeal clades, the remainder (yellow) are bacterial. Error bars: 2×SEM. Digits below bars indicate the numbers of orthologous groups. (*B*) Values as in (*A*), paired and without error bars. Red-dashed: results with higher mean proportion of sharing species for MP than WS. Yellow-dotted: results with MP<WS but *P*-value over a cutoff of 0.05 in a two-sample Welch *t*-test. Green-solid: results with *P*<0.05.

The results show that the mean numbers of species sharing each of these ancestral OGs are lower for membrane-bound than for water-soluble proteins across 31 of the 35 clades studied (fig. 7; paired *t*-test: *t* = 7.31; d*f* = 34; *P* = 1.81·10^−8^; *r* = 0.782). That is, membrane proteins have been lost more often than water-soluble proteins between closely related taxa, confirming that true gene loss can also account in part for the lower homology of membrane proteins reported here.

## Discussion

We report that membrane proteins have fewer orthologs than water-soluble proteins across the entire tree of life (figs. 2 and 4). In principle this finding could be due to a higher evolutionary rate, which prevents sequence-searching algorithms such as BLAST from detecting homologs beyond a given threshold, or it could correspond to true gene loss. We show that both mechanisms are at play. First, we demonstrate that evolutionary rates are faster for membrane proteins than for water-soluble proteins across the whole tree of life, and in each of the three domains of archaea, bacteria, and eukaryotes independently (fig. 5*A–D*). Significantly, the evolutionary rates of membrane proteins are faster in the outside-facing aqueous regions than in their inside-facing counterparts (fig. 5*E* and supplementary figs. S2–S4, Supplementary Material online). Second, our analysis of closely related species shows that predicted ancestral proteins have been lost more frequently if they were membrane bound (fig. 7). This indicates that the lower homology of membrane proteins is not only due to divergence beyond sequence recognition, but also that true gene loss has occurred.

It has been reported that exported water-soluble proteins evolve faster than cytosolic proteins, and indeed faster than the external sections of membrane proteins in mammals (Julenius and Pedersen 2006). Our hypothesis predicts that a similar pattern of low homology and greater gene loss should be observed for excreted water-soluble proteins. We confirm that this is indeed the case (fig. 6*A* and *B*), with universality decreasing the further out the cell from cytosolic, to membrane-bound, to extracellular proteins.

Membrane-bound and extracellular proteins are in general less central to metabolic networks and functions (Julenius and Pedersen 2006), so the patterns we report could be caused by stronger selective constraints operating on cytosolic proteins, and a comparatively relaxed evolution of more peripheral proteins. However, at least for mammals, the faster evolutionary rates of membrane-bound and extracellular proteins do not seem to depend on the essentiality of the proteins themselves (Liao et al. 2010), suggesting that mechanisms other than purifying selection on purportedly less crucial membrane-bound and exported proteins are at play.

Our findings suggest the operation of a more general evolutionary principle: membrane proteins evolve faster because they face stronger adaptive selection in changing environments, whereas cytosolic proteins are under more stringent purifying selection in the homeostatic interior of the cell (fig. 1). The outside-facing sections of membrane-spanning proteins are closely involved in adaptation to new environments and functions, and so are more likely to diverge over time than the cytosolic portions. As emerging species colonize novel environments or specialize in new tasks, the outside-facing sections are subject to stronger positive selection, whereas rate-limiting purifying selection prevails in the membrane-spanning and inside-facing portions (fig. 5*E* and *F*; supplementary figs. S2–S4, Supplementary Material online). Novel or changing environments are also likely to reduce the utility of existing membrane proteins, leading to loss over time, and accounting for the absences that we observe in closely related species (fig. 7). Our hypothesis immediately suggests that this effect should be strongest in prokaryotes, weaker in unicellular eukaryotes (where intracellular organelles can provide an additional homeostatic environment for membrane proteins), and weakest in multicellular eukaryotes (where even extracellular proteins face a homeostatic environment provided by tissues and organs). That is indeed the case (fig. 3*A*), although the difference in size of ortholog groups between membrane proteins and water-soluble proteins remains substantial even in multicellular eukaryotes. Moreover, the difference between water-soluble and membrane-bound proteins is greater for proteins most simply assumed to be ancient to eukaryotes (fig. 3*B*). This reinforces the suggestion that ancient membrane-bound proteins are more likely to either diverge beyond recognition or be lost entirely than their water-soluble counterparts.

This broad evolutionary perspective provides a framework for interpreting a number of earlier findings that have proved difficult to generalize. Previous results show that water-soluble proteins secreted from the cell evolve faster than cytosolic proteins in mammals and yeast, and that the external portions of membrane proteins evolve faster than the internal domains (Julenius and Pedersen 2006). However, given the complexity of mammalian species, a focus on this taxonomic class does not lend itself to generalizations about purifying selection or adaptation to changing extracellular environments. Similarly, the G-protein-coupled receptor superfamily is known to evolve faster in its extracellular portions than in the transmembrane and cytosolic regions, but this has again been interpreted in terms of particular functional and structural constraints (Tourasse and Li 2000; Lee et al. 2003). In Gram-negative bacteria, degradation of xenobiotic toxic substances occurs in the periplasmic space (Kawai 1999; Nagata et al. 1999), making evolutionary pressure stronger on the external regions than in the homeostatic interior. Signal peptides have been shown to evolve rapidly in both prokaryotes and eukaryotes, pointing to positive selection on these secretory membrane-targeting fragments (Li et al. 2009). Finally, parasitic interactions can promote the rapid evolution of membrane proteins, especially the external loops involved directly in antigen interactions (Volkman et al. 2002; Plotkin et al. 2004). Parasite membrane proteins face positive selective pressure from recognition by the host, but these red-queen dynamics have not been extended beyond parasite–host interactions. We show that each of these specific instances can be generalized for membrane proteins as a class across the tree of life. When interpreted in a more comprehensive context, all these observations point to faster evolution outside the cell in response to changing environments or functions.

We have not considered the effects of horizontal gene transfer (HGT), a major force in microbial evolution, as the unequivocal detection and ecological significance of ancient HGT events is still a hotly debated topic (Philippe and Douady 2003; Dagan and Martin 2007; Koonin 2014, 2015; Puigbò et al. 2014; Akanni et al. 2015; Boothby et al. 2015; Katz 2015; Ravenhall et al. 2015; Soucy et al. 2015; Koutsovoulos et al. 2016). Horizontally transferred genes tend to be integrated at the periphery of metabolic networks, whereas genes at the core tend to be more evolutionarily conserved (Pál et al. 2005). As noted earlier, at the level of cellular gene networks, extracellular proteins could be considered peripheral, whereas intracellular proteins are more central, and so should be more conserved (Julenius and Pedersen 2006). But our data suggest that membrane proteins are not more likely to be lost (or horizontally transferred) simply because they are peripheral to gene networks. The observation that the outside-facing portions of membrane proteins evolve faster than their cytosolic counterparts (fig. 5*E* and *F*), and that the greater the extracellular content the less widely conserved the protein (fig. 6*A* and *B*), are more consistent with selection operating differently outside the cell.

We conclude that adaptation to novel environments and functions underlies the lower homology of membrane proteins across the tree of life. Life is defined by its cellular nature: the inside of a living cell is separated from its environment by an organic membrane. Cells must constantly interact with varying environments, while maintaining tight internal homeostasis. The interactions between the inside and outside of the cell are largely mediated by membrane-bound and exported proteins, so elucidating their evolution is central to understanding the origins and evolution of life. For the same reasons, membrane proteins have great medical importance. Over half of all known drug targets are membrane proteins, so our findings may help to explain why the progression of new drugs from animal models into human trials is so often unsuccessful (Holmes et al. 2011; Denayer et al. 2014). Our results are also of practical importance in phylogenetics: if membrane proteins are less than half as likely to be conserved widely across the tree of life, then homology searches will often be confounded, as could molecular clocks. Faster evolution outside the cell makes simple intuitive sense, but the strength of this signal across the whole tree of life elevates what has been seen as an interesting sporadic pattern into a general principle of evolution.

## Materials and Methods

### Acquisition of Orthologs

The full set of ortholog groups (OGs) from the OMA database was downloaded from the OMA server at www.omabrowser.org/export, September 2014 release.

The species in the list of reference proteomes for figure 2 were obtained from EMBL-EBI at www.ebi.ac.uk/reference_proteomes (last accessed August 8, 2016). Two of the 66 species in the list were not found on OMA (supplementary table S1, Supplementary Material online). For each species, in figure 2, the downloaded OMA data set was scanned for any OGs containing the species of interest, and the size of the OG determined (i.e., the total number of orthologs in the group, or equivalently the number of species with an identifiable ortholog). This implies that several OGs were counted multiple times, i.e., if they were shared by two or more of the 64 species in the data set. Removing these duplications had no effect on our conclusions (data available upon request).

The set of 883,176 OMA OGs includes multiple orthologs shared by multiple strains of the same species (e.g., *Escherichia coli*), so, as a strategy to avoid oversampling in the phylogenetic-distribution analysis of figure 4, one sequence was chosen per clade at the sixth level of taxonomic differentiation according to the NCBI taxonomy browser. As an example, the full NCBI taxonomic lineage for *E. coli* is 1.Bacteria > 2.Proteobacteria > 3.Gammaproteobacteria >  4.Enterobacteriales > 5.Enterobacteriaceae > 6.Escherichia >  7.*Escherichia coli*, from where the sixth taxonomic level is “Escherichia”; similarly, the equivalent for humans is “Deuterostomia”, from: 1.Eukaryota > 2.Opisthokonta >  3.Metazoa > 4.Eumetazoa > 5.Bilateria > 6.Deuterostomia. Only OGs with 3 or more different such clades were kept. This left a total of 228,148 OGs. When multiple sequences were found for the same clade, as defined earlier, a representative was chosen from well-annotated species (e.g., *Escherichia coli*, *Saccharomyces cerevisiae*, *Homo sapiens*, *Methanosarcina acetivorans*), where available, or at random.

Since each protein was classified in a binary fashion as either WS or MP (except for the analyses in fig. 6), the logistic regressions of figure 4 were produced by fitting a quasi-binomial model to the type of protein (0 for WS and 1 for MP), using as predictor the number of orthologs in the cluster (i.e., the size of the OMA OG, or more simplistically the number of taxa that have an identifiable ortholog of the protein in OMA). The points were produced entirely independently by binning the data in 10% increments of how many taxa share each protein, or size of the OG. That is, for figure 4*A*, the total number of taxa is 489, so proteins in the first bin are shared by between 3 and 49 clades. The point represents the proportion of those proteins that are MPs.

### BLAST Searches

The full proteomes of six representative species for supplementary fig. S1, Supplementary Material online, were procured from the EMBL-EBI list of reference proteomes. The complete nonredundant (nr) protein database was downloaded from NCBI. The BLASTp algorithm and software was also downloaded from NCBI and run locally for each protein in each of the six selected proteomes. Significant BLASTp matches were defined as having an *e*-value <10^−10^ and a query coverage of at least 70%; when multiple hits were found for the same species, only one hit was counted.

Separately, a BLAST-able database was created from all sequences in the 228,148 selected OMA ortholog groups using the makeblastdb command in the NCBI BLAST suite. BLASTp was then used to detect hits of PDB sequences against this local OMA OGs database for figure 5*E*; the ortholog group containing the best hit was found, aligned and sliced into portions as described below in “Estimation of evolutionary rates”. Whenever two PDBs retrieved the same OG as their best hit, the one with the best alignment (judged by Nei’s sequence-diversity measure, π) was kept.

### Identification of Membrane Proteins

Membrane proteins were annotated using the predictions of the TMHMM 2.0c algorithm (Krogh et al. 2001). This algorithm predicts only trans-membrane alpha helical proteins, so on preliminary tests undetected ortholog groups marked as porins or integral membrane proteins in their descriptions in OMA were further annotated as MPs. Gene Ontology annotations, where available, were used in addition to identify further MPs in these preliminary tests. All other proteins were assumed to be WS. These additional classifications of MPs produced only minor changes that did not alter our conclusions (data available upon request), so to ensure reproducibility and avoid unpredictable effects of the sparse annotations, for all results in the article MPs were annotated using only the predictions of the TMHMM algorithm.

### Identification and Analysis of Extracellular Proteins and Regions

Extracellular (exported) proteins for analyses in figure 6 were identified using SignalP 4.1 (Petersen et al. 2011). This software cannot presently provide reliable predictions of signal peptides in archaea or bacterial Tenericutes, so results for these were ignored. If the majority of orthologs within an ortholog group were identified positively by SignalP, the OG was considered to contain a signal peptide. These classifications were used to separate water-soluble proteins into cytosolic and exported for figure 6*A* and *C*. For figure 6*B*, the proportion of extracellular residues was computed for membrane proteins by parsing the predictions of TMHMM discussed earlier. For each orthologous group, the lengths of outside-facing residues in proteins predicted as containing trans-membrane helices were summed and divided over the sum of the total sequence lengths of the same proteins. This proportion was assigned to the corresponding ortholog group, and the data binned in 10% intervals as shown in figure 6*B*. In addition, water-soluble proteins predicted by the SignalP analysis as cytosolic (negative results) were added to the first bin (0.0–0.1), whereas predicted extracellular proteins were added to the last bin (0.9–1.0). The points were then computed by averaging the ortholog-group sizes within each bin. The predictions of SignalP were only used for figure 6 and its related analyses. In all other analyses in the article we considered only MPs versus WSs in general.

### Estimation of Evolutionary Rates and Selective Constraints. Slicing of Membrane-Protein Portions

For figure 5*A*–*D*, the protein sequences of each of the 228,148 OMA OGs shared by three or more clades were aligned using MAFFT (Katoh and Standley 2013). Nei’s sequence-diversity measure (π, Nei and Li 1979) was calculated by averaging the number of differences per alignment position per pair of sequences, and then averaging these over the number of pairs, for each group of orthologs. These values were used in figure 5*A*–*D* and in the re-classifications of water-soluble proteins as extracellular versus intracellular in figure 6*C*. Nei’s π was computed in the same manner for figure 5*E* and supplementary figure S3, Supplementary Material online.

**Fig. 8:**
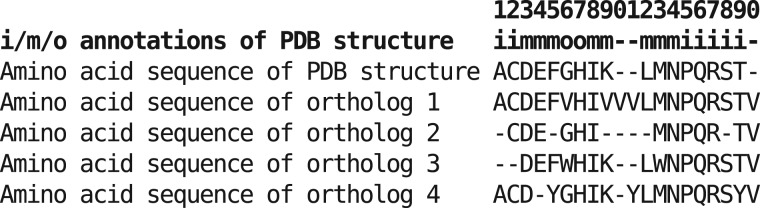
An example of the alignment slicing process. Here, *i*, *m*, and *o* represent that the corresponding amino acid in the PDB structure is annotated as inside, membrane-spanning, or outside (respectively), either in the PDBTM database for figure 5*E*, or in the 12 annotations created by directly inspecting the PDB structure against the primary literature for supplementary figures S3 and S4, Supplementary Material online. In the example here, positions 1–2, and 15–19 are inside; 6–7 are outside; 3–5, 8–9, 25 and 12–14 are membrane-spanning; and 10–11, 20 are not present in the reference sequence and are therefore ignored.

For results in figure 5*E*, the entire nonredundant set of PDB amino-acid sequences and annotations was downloaded from pdbtm.enzim.hu (Tusnády et al. 2004). This data set is constantly updated to include all PDB structures for membrane proteins in the PDB database, and the files parsed into annotations for the subcellular localization of each amino acid in each of these structures, where the information is available (often the crystal structures have unresolved portions, notably loops, and in other cases the researchers do not report whether an aqueous section is inside- or outside-facing, in which case the protein was ignored altogether). At the time of this analysis there were 576 nonredundant integral membrane proteins in PDBTM (496 annotated as alpha helices and 80 as beta barrels), 378 of which unambiguously specified inside- versus outside-facing aqueous regions. Homologs were procured by BLASTp search against a local subset of the OMA database created as described earlier, and alignments produced with MAFFT. To “slice” (i.e., split vertically) the multiple-sequence alignments (MSAs) used in figure 5*E* and supplementary figures S2–S4, Supplementary Material online, into the membrane-spanning, inside, outside, and aqueous (which includes both inside- and outside-facing) sections, the PDBTM annotations (fig. 5*E* and supplementary fig. S2, Supplementary Material online) or the hand-annotated positions (supplementary figs. S3 and S4, Supplementary Material online) for the reference PDB protein sequence were used to establish the sub-cellular location of each amino acid. Each position was then sliced as described in the toy example in figure 8. The “aqueous” portions were constructed by concatenating the inside and outside alignments.

As an additional measure to confirm our results, we built trees using FastTree 2.0 (Price et al. 2010) with default options (Jones–Taylor–Thornton model with CAT approximation) for the full alignment and for each of the three sliced portions, and computed the mean of the branch lengths of all nodes within each tree. The branch lengths in a tree correspond to the number of substitutions per site, so we used their mean as a further estimate of evolutionary rate in supplementary figures S2 and S4, Supplementary Material online. Branch lengths were extracted directly from the FastTree output trees computed on the whole and sliced MAFFT alignments.

### Analysis of Closely Related Species

Due to the difficulty of reliably inferring phylogenetic trees spanning all three domains of life, as well as widespread horizontal gene transfer within and across the domains, we used a coarse but robust approach to infer genes present in the common ancestor of closely related taxa: clades with 10 or more closely related species or strains of archaea and bacteria were detected by exploring the NCBI taxonomy of species included in OMA (omabrowser.org/oma/export/, last accessed August 8, 2016). These clades of “closely related” species, in figure 7, were defined as sharing members at the fifth or higher taxonomic level of differentiation, according to the NCBI lineages (as earlier). The pre-computed OMA data set was then scanned for any OGs containing members of each of these clades. OGs shared by more than half of the members of the clade were considered ancestral, and the rest were ignored. These ancestral OGs were then classified as either MPs or WSs using a majority census for the predictions of the TMHMM algorithm on each sequence in the group.

### Bioinformatics

BioPython (Cock et al. 2009), ETE (Huerta-Cepas et al. 2010), TMHMM (Krogh et al. 2001), SignalP (Petersen et al. 2011), and R (R Core Team 2014) were used widely in the calculations and analyses in this article. Data can be accessed at dx.doi.org/10.5061/dryad.00731.

## Supplementary Material

Supplementary tables S1 and S2 and figures S1–S4 are available at *Molecular Biology and Evolution* online (http://www.mbe.oxfordjournals.org/).

Supplementary Data
