# Supplementary material for: Membrane Proteins Are Dramatically Less Conserved than Water-Soluble Proteins across the Tree of Life
Source: Mol Biol Evol. 2016 Aug 8;33(11):2874–84. doi: 10.1093/molbev/msw164 (PMC5062322; doi:10.1093/molbev/msw164)
Supplement: Supplementary Data [file supp_33_11_2874__index.html]

Membrane proteins are dramatically less conserved than water-soluble proteins across the tree of life — Membrane Proteins Are Dramatically Less Conserved than Water-Soluble Proteins across the Tree of Life — Membrane Proteins Are Dramatically Less Conserved than Water-Soluble Proteins across the Tree of Life — Supplementary Data 

# Membrane Proteins Are Dramatically Less Conserved than Water-Soluble Proteins across the Tree of Life

## Supplementary Data

files

- Supplementary Data - docx file
