# Supplementary material for: Membrane Proteins Are Dramatically Less Conserved than Water-Soluble Proteins across the Tree of Life
Source: Mol Biol Evol. 2016 Aug 8;33(11):2874–84. doi: 10.1093/molbev/msw164 (PMC5062322; doi:10.1093/molbev/msw164)
Supplement: Supplementary Data [file supp_msw164_MBE-16-0285_Lane_Supplementary_Information.docx]

**Supplementary Information**

**Membrane proteins are dramatically less conserved than water-soluble proteins across the tree of life**

Victor Sojo^1,2,3^, Christophe Dessimoz^2,4,5^, Andrew Pomiankowski^1,2^, Nick Lane^1,2^

^1^CoMPLEX & ^2^Department of Genetics, Evolution and Environment. University College London. Gower Street, London, WC1E 6BT, U.K.

^3^Systems Biophysics, Faculty of Physics, Ludwig-Maximilian University of Munich. Amalienstr. 54. 80799 Munich, Germany.

^4^Dept. of Ecology and Evolution & ^5^Center for Integrative Genomics. University of Lausanne. Biophore 4309, 1015 Lausanne, Switzerland.

**Corresponding authors:** Victor Sojo or Nick Lane

**Address:** Department of Genetics, Evolution and Environment. Darwin Building, Room 610. University College London. Gower Street, London WC1E 6BT, United Kingdom.

**email:** [v.sojo.11@ucl.ac.uk](mailto:v.sojo.11@ucl.ac.uk) or nick.lane@ucl.ac.uk

**Phone:** +44 (0) 20 7679 1385

# Supplementary Figures


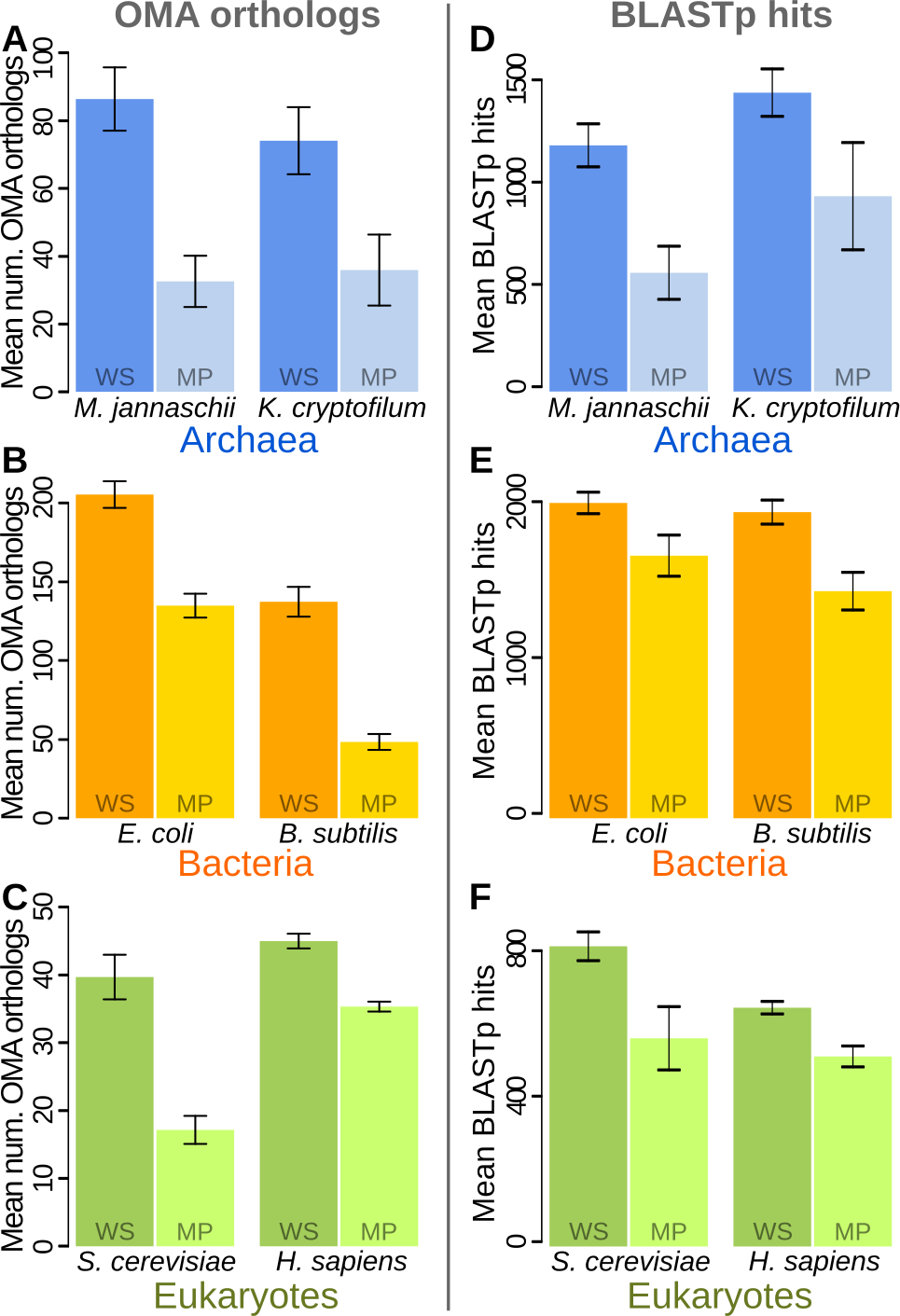


Fig. S1. blastp search confirms lower homology of membrane proteins.

A blastp search on the non-redundant (nr) NCBI protein database confirms the results in fig. 2 that membrane-bound proteins recover fewer homologs. Two well-annotated representative species from distant clades of each domain were chosen, namely a euryarchaeon and a TACK-archaeon, a Gram-positive and a Gram-negative bacterium, and a unicellular and a multicellular eukaryote. Results from OMA in fig. 2 are repeated in (**A**, **B** and **C**), for comparison with blastp results in (**D**, **E** and **F**). In all cases the mean number of blastp hits is lower for MPs than for WSs. Bars represent the means ± 2SEM.


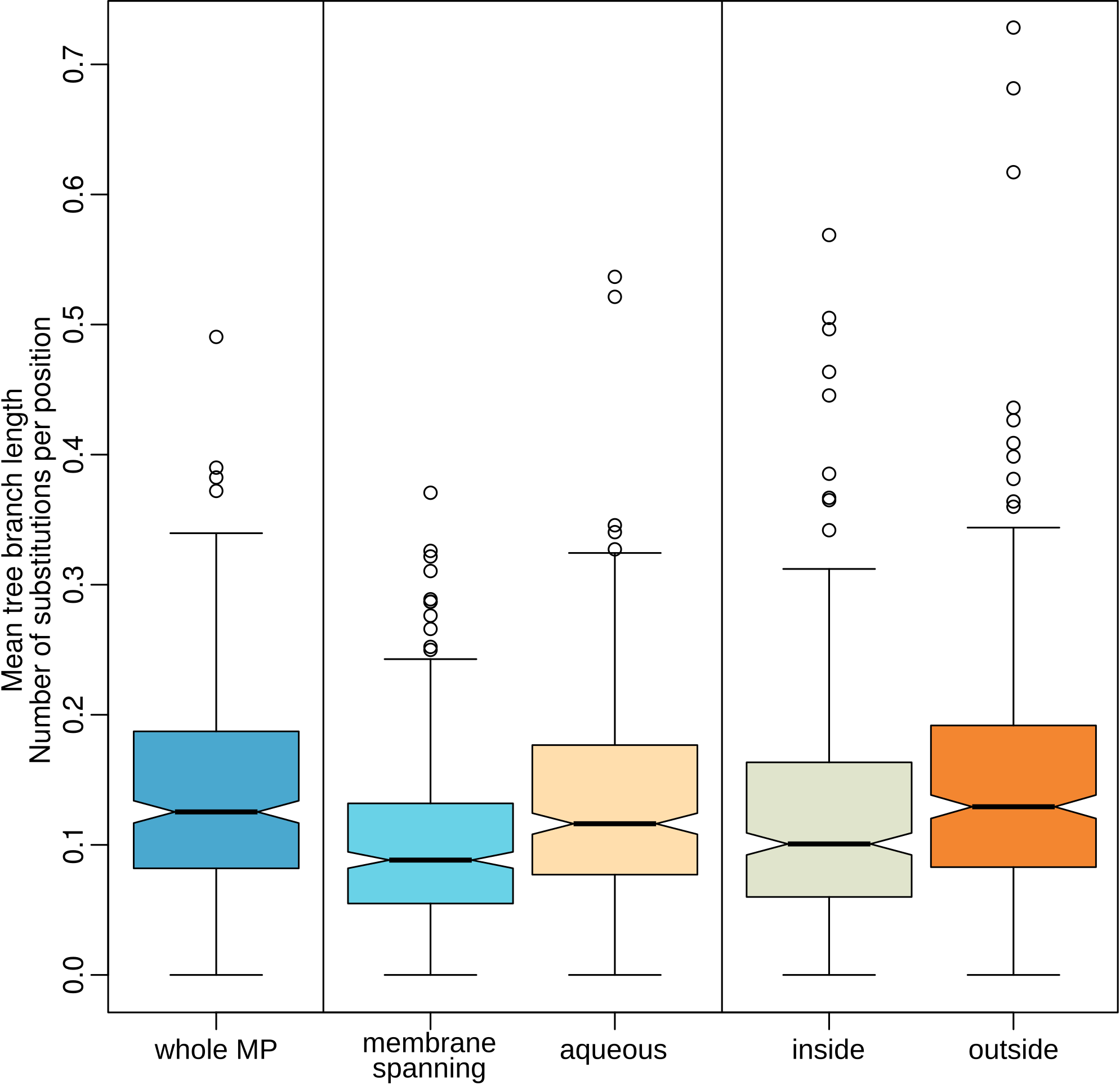


Fig. S2. Mean tree branch lengths confirm results in fig. 5E.

The mean branch lengths of trees built from the whole alignments and sliced portions confirm that aqueous regions of membrane proteins evolve faster than membrane-spanning regions. Amongst the aqueous sections, both of which evolve faster than the membrane-spanning sections overall, the outside-facing ones evolve faster than their inside-facing counterparts. This confirms the results in fig. 5E.


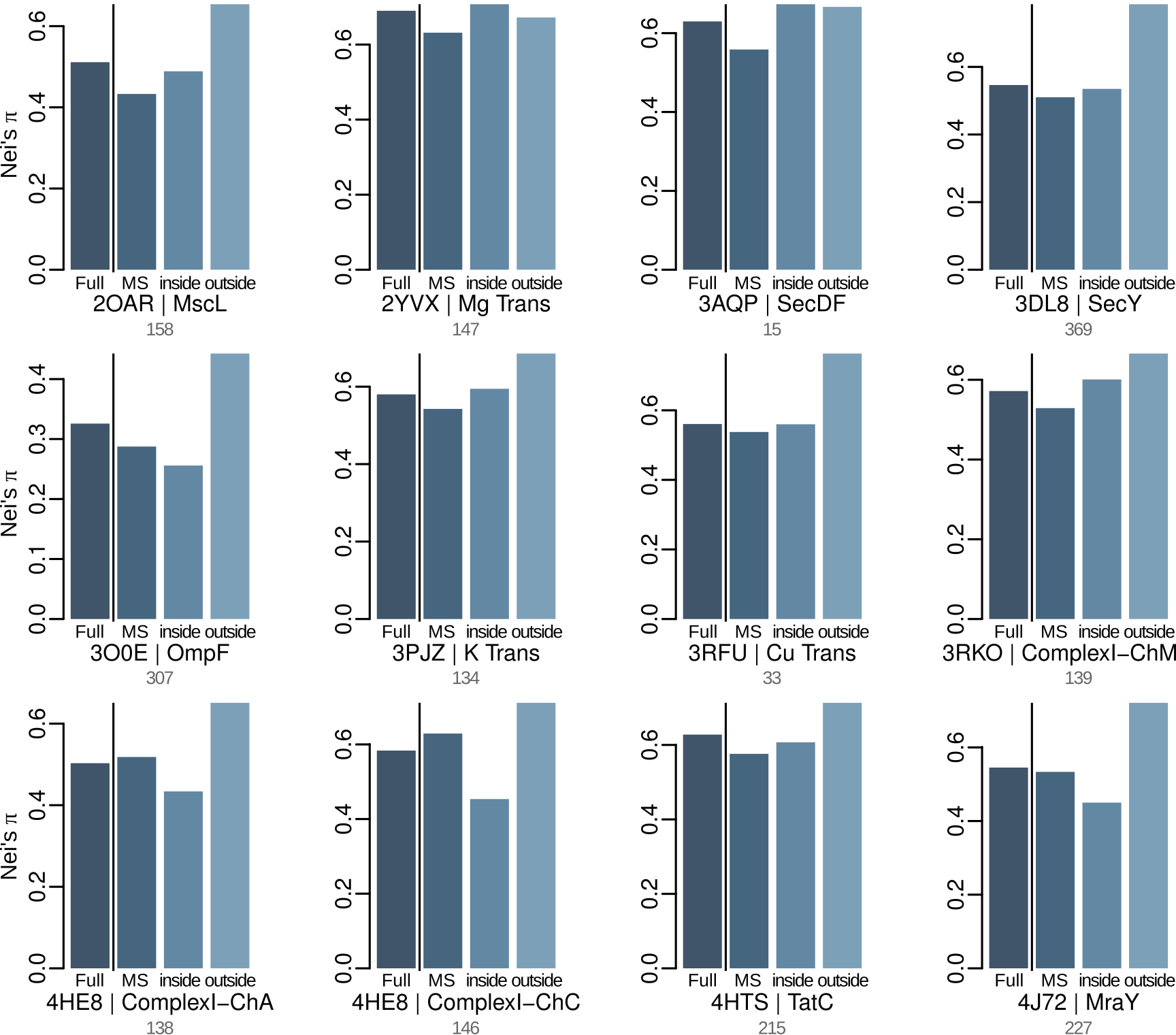


Fig. S3. Nei’s π shows that outside-facing sections of membrane proteins typically evolve faster than inside-facing sections.

In ten of the twelve proteins annotated by visually inspecting the PDB structure in relation to the original literature, evolution occurs faster for outside-facing than for inside-facing aqueous sections. This occurs both in outer-membrane and inner-membrane proteins. Four-character codes (e.g. “4HE8”) represent the PDB entry of the protein, followed by a short description of the protein name or function, as per the original literature. Full: the whole multiple-sequence alignment, without slicing. MS: membrane-spanning section (i.e. the lipid-exposed or “middle” section of a trans-membrane protein). Details of proteins and primary references in supplementary table S2. Digits under the protein names indicate the number of sequences used in the comparison. See confirmation of these results in supplementary fig. S4.


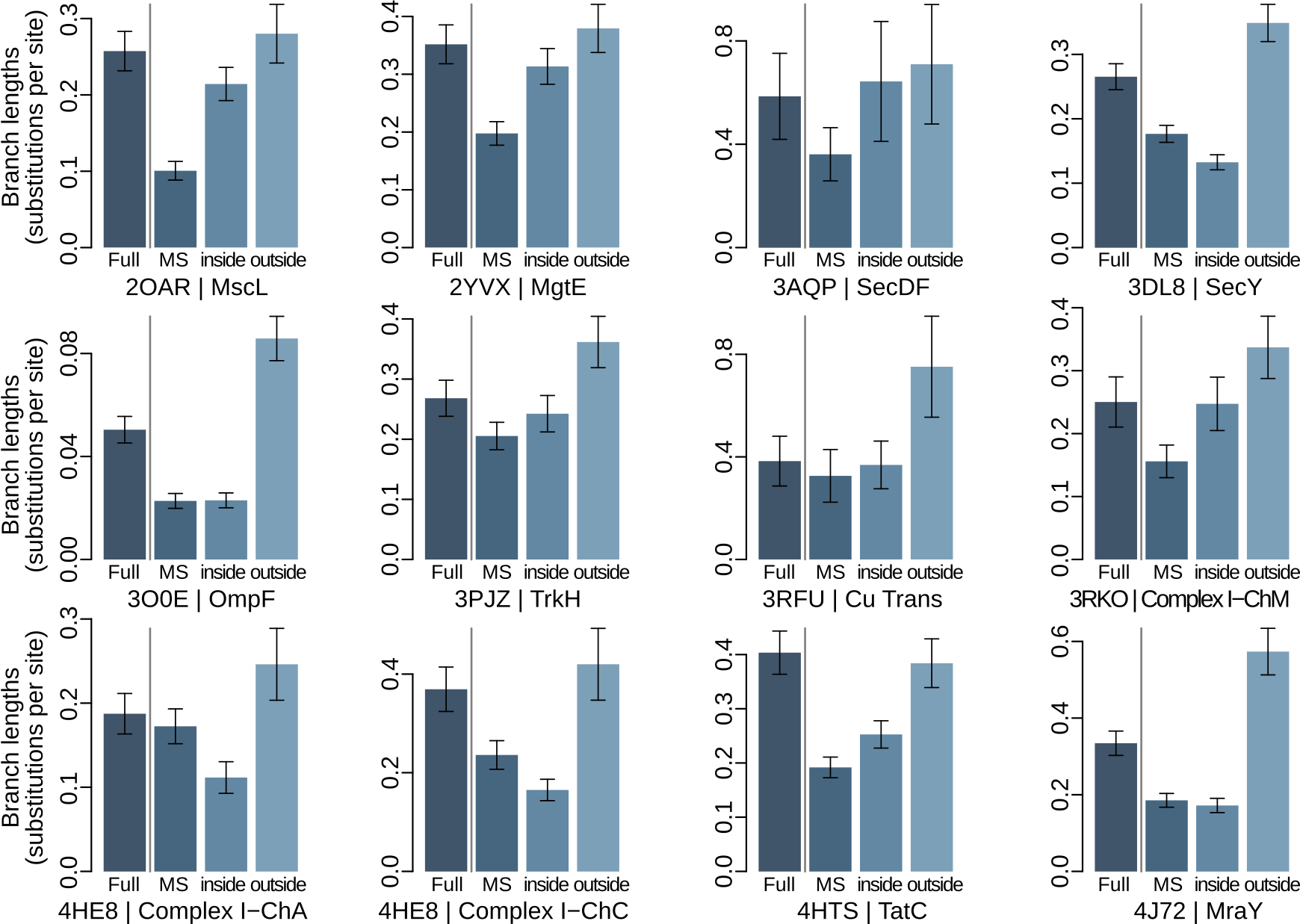


Fig. S4. Mean tree branch lengths confirm results in supplementary fig. S3.

Computing mean branch lengths of trees for each portion as described in the final paragraph of the Evolutionary Rates section in the Materials and Methods confirms that evolution occurs faster for outside-facing than for inside-facing aqueous sections. Under this analysis, all estimates show the outside sections evolving faster than their inside counterparts. Bars represent the means of the branch lengths of all nodes in each tree ± 2SEM. Details as in supplementary fig. S3.

# Supplementary Tables

Table S1. The mean size of an OMA ortholog group is smaller for membrane proteins in all cases.

| **Species**^a^ | **OMA**  **code**^b^ | **Num. proteins in OMA** | **Num.**  **MPs** | **Proportion of MPs** | **Mean WS OG size** | **Mean MP OG size** |
| --- | --- | --- | --- | --- | --- | --- |
| **Archaea** |  |  |  |  |  |  |
| *Halobacterium salinarum* | HALSA | 2241 | 472 | 0.211 | 66.2 | 25.9 |
| *Korarchaeum cryptofilum* | KORCO | 1192 | 213 | 0.179 | 74.1 | 35.9 |
| *Methanosarcina acetivorans* | METAC | 1639 | 285 | 0.174 | 86.4 | 32.6 |
| *Methanocaldococcus jannaschii* | METJA | 3514 | 878 | 0.250 | 60.6 | 26.8 |
| *Sulfolobus solfataricus* | SULSO | 2668 | 578 | 0.217 | 62.8 | 29.4 |
| *Thermococcus kodakarensis* | THEKO | 2039 | 469 | 0.230 | 70.1 | 26.5 |
| **Bacteria** |  |  |  |  |  |  |
| *Aquifex aeolicus* | AQUAE | 1393 | 271 | 0.195 | 216.5 | 74.7 |
| *Bacillus subtilis* | BACSU | 3984 | 1122 | 0.282 | 137.3 | 48.4 |
| *Bacteroides thetaiotaomicron* | BACTN | 3931 | 974 | 0.248 | 98.5 | 38.7 |
| *Bradyrhizobium japonicum*^c^ | BRAJA | 6937 | 1723 | 0.248 | 85.2 | 41.7 |
| *Chloroflexus aurantiacus* | CHLAA | 3802 | 1104 | 0.290 | 123.1 | 38.4 |
| *Chlamydia trachomatis* | CHLTR | 889 | 219 | 0.246 | 243.6 | 67.7 |
| *Deinococcus radiodurans* | DEIRA | 2519 | 478 | 0.190 | 138.5 | 59.1 |
| *Dictyoglomus turgidum* | DICTD | 1673 | 454 | 0.271 | 204.2 | 43.6 |
| *Escherichia coli* | ECOLI | 4264 | 1045 | 0.245 | 205.4 | 134.9 |
| *Fusobacterium nucleatum* | FUSNN | 1661 | 352 | 0.212 | 181.9 | 66.7 |
| *Geobacter sulfurreducens* | GEOSL | 3066 | 823 | 0.268 | 154.2 | 52.6 |
| *Gloeobacter violaceus* | GLOVI | 3380 | 696 | 0.206 | 101.5 | 38.0 |
| *Leptospira interrogans* | LEPIN | 3645 | 1031 | 0.283 | 106.9 | 35.3 |
| *Mycobacterium tuberculosis* | MYCTU | 3933 | 797 | 0.203 | 123.2 | 56.5 |
| *Pseudomonas aeruginosa* | PSEAE | 5500 | 1327 | 0.241 | 135.5 | 79.7 |
| *Rhodopirellula baltica* | RHOBA | 3218 | 745 | 0.232 | 106.4 | 27.2 |
| *Streptomyces coelicolor* | STRCO | 7104 | 1719 | 0.242 | 72.7 | 25.8 |
| *Synechocystis sp.* | SYNY3 | 3058 | 739 | 0.242 | 139.4 | 48.7 |
| *Thermotoga maritima* | THEMA | 1779 | 431 | 0.242 | 185.7 | 53.8 |
| *Thermodesulfovibrio yellowstonii* | THEYD | 1716 | 393 | 0.229 | 211.4 | 77.3 |
| **Eukaryota (unicellular)** |  |  |  |  |  |  |
| *Aspergillus fumigatus*^d^ | ASPFU | 8801 | 1826 | 0.207 | 41.2 | 23.1 |
| *Candida albicans*^e^ | CANAW | 4932 | 949 | 0.192 | 40.3 | 18.7 |
| *Cryptococcus neoformans* | CRYNJ | 5679 | 1094 | 0.193 | 39.5 | 19.1 |
| *Giardia intestinalis* | GIAIC | 1181 | 211 | 0.179 | 25.5 | 6.0 |
| *Leishmania major* | LEIMA | 7858 | 1423 | 0.181 | 14.1 | 6.7 |
| *Monosiga brevicollis* | MONBE | 4184 | 775 | 0.185 | 43.9 | 16.4 |
| *Phaeosphaeria nodorum* | PHANO | 15023 | 2601 | 0.173 | 23.3 | 16.2 |
| *Plasmodium falciparum* | PLAF7 | 1853 | 375 | 0.202 | 33.6 | 11.8 |
| *Schizosaccharomyces pombe* | SCHPO | 3541 | 602 | 0.170 | 62.3 | 24.7 |
| *Yarrowia lipolytica* | YARLI | 4222 | 862 | 0.204 | 57.9 | 22.7 |
| *Saccharomyces cerevisiae* | YEAST | 4811 | 926 | 0.192 | 39.7 | 17.2 |
| **Eukaryota (multicellular)** |  |  |  |  |  |  |
| *Anopheles gambiae* | ANOGA | 9889 | 2390 | 0.242 | 33.8 | 17.5 |
| *Arabidopsis thaliana* | ARATH | 23989 | 6102 | 0.254 | 21.1 | 12.1 |
| *Bos taurus* | BOVIN | 19336 | 5432 | 0.281 | 46.5 | 31.9 |
| *Branchiostoma floridae* | BRAFL | 15318 | 3050 | 0.199 | 23.0 | 14.0 |
| *Caenorhabditis elegans* | CAEEL | 15719 | 5280 | 0.336 | 21.5 | 8.4 |
| *Canis familiaris* | CANFA | 18574 | 4805 | 0.259 | 45.0 | 34.7 |
| *Gallus gallus* | CHICK | 14226 | 3535 | 0.248 | 47.8 | 35.7 |
| *Ciona intestinalis* | CIOIN | 9346 | 1932 | 0.207 | 31.9 | 15.3 |
| *Danio rerio* | DANRE | 22138 | 5805 | 0.262 | 30.4 | 20.8 |
| *Dictyostelium discoideum* | DICDI | 8316 | 1793 | 0.216 | 25.2 | 8.9 |
| *Drosophila melanogaster* | DROME | 13582 | 3454 | 0.254 | 30.3 | 16.9 |
| *Homo sapiens* | HUMAN | 20221 | 5242 | 0.259 | 45.0 | 35.4 |
| *Ixodes scapularis* | IXOSC | 8470 | 1722 | 0.203 | 27.0 | 15.7 |
| *Macaca mulatta* | MACMU | 19771 | 4671 | 0.236 | 39.7 | 34.3 |
| *Monodelphis domestica* | MONDO | 18904 | 5168 | 0.273 | 40.6 | 28.7 |
| *Mus musculus* | MOUSE | 20457 | 6047 | 0.296 | 45.0 | 29.5 |
| *Nematostella vectensis* | NEMVE | 14935 | 2705 | 0.181 | 25.7 | 14.6 |
| *Neurospora crassa* | NEUCR | 6817 | 1241 | 0.182 | 35.4 | 22.8 |
| *Ornithorhynchus anatinus* | ORNAN | 14308 | 3247 | 0.227 | 31.7 | 23.4 |
| *Pan troglodytes* | PANTR | 18241 | 4551 | 0.249 | 46.5 | 37.3 |
| *Physcomitrella patens* | PHYPA | 13463 | 3126 | 0.232 | 25.6 | 13.1 |
| *Rattus norvegicus* | RATNO | 20502 | 5872 | 0.286 | 42.7 | 29.0 |
| *Schistosoma mansoni* | SCHMA | 4023 | 745 | 0.185 | 34.4 | 19.0 |
| *Sclerotinia sclerotiorum* | SCLS1 | 8377 | 1576 | 0.188 | 34.2 | 18.7 |
| *Takifugu rubripes* | TAKRU | 17576 | 4480 | 0.255 | 36.0 | 24.3 |
| *Ustilago maydis* | USTMA | 3505 | 690 | 0.197 | 43.8 | 17.2 |
| *Xenopus tropicalis* | XENTR | 16021 | 4184 | 0.261 | 31.6 | 21.4 |

^a^ Two of the 66 species, namely *Thalassiosira pseudonana* (THAPS) and *Trichomonas vaginalis* (TRIVA), were not found in OMA at the time of this analysis and were thus ignored.

^b^ Five-character codes correspond to those used in fig. 2 and supplementary fig. S1. Species are the 66 organisms in the EMBL-EBI’s list reference proteomes.

^c^ *Bradyrhizobium diazoefficiens* (BRADU) is in the EBI Reference Proteomes list, but was not found in OMA at the time of this analysis; *B. japonicum* (BRAJA) was used instead.

^d^ In OMA as “*Neosartorya fumigata*” at the time of writing.

^e^ The OMA code for the *Candida albicans* strain used was (CANAW) instead of the one in the EBI list (CANAL), not found in OMA at the time of this analysis.

Table S2

PDB entry codes (3-D structures deposited on rcsb.org), descriptions, and references in the primary literature for the proteins in supplementary figs. S3 and S4.

| **PDB entry** | **Short description** | **Long description** | **Reference** |
| --- | --- | --- | --- |
| 2OAR | MscL | Mechanosensitive channel of large conductance | (Chang et al. 1998) |
| 2YVX | MgtE | Magnesium transporter | (Hattori et al. 2007) |
| 3AQP | SecDF | Translocon-associated membrane protein | (Tsukazaki et al. 2011) |
| 3DL8 | SecY | Bacterial protein translocation channel | (Zimmer et al. 2008) |
| 3O0E | OmpF | Bacterial outer-membrane porin | (Housden et al. 2010) |
| 3PJZ | TrkH | Potassium transporter | (Cao et al. 2011) |
| 3RFU | Cu Trans | Cu-transporting PIB-type ATPase | (Gourdon et al. 2011) |
| 3RKO | Complex I-M | Chain M of respiratory complex I | (Efremov and Sazanov 2011) |
| 4HE8 | Complex I-A | Chain A of respiratory complex I | (Baradaran et al. 2013) |
| 4HE8 | Complex I-C | Chain C of respiratory complex I | (Baradaran et al. 2013) |
| 4HTS | TatC | Twin arginine translocase receptor | (Ramasamy et al. 2013) |
| 4J72 | MraY | Polyprenyl-phosphate N-acetyl hexosamine 1-phosphate transferase | (Chung et al. 2013) |

## Supplementary References

Baradaran R, Berrisford JM, Minhas GS, Sazanov LA. 2013. Crystal structure of the entire respiratory complex I. *Nature* 494:443–448.

Cao Y, Jin X, Huang H, Derebe MG, Levin EJ, Kabaleeswaran V, Pan Y, Punta M, Love J, Weng J, et al. 2011. Crystal structure of a potassium ion transporter, TrkH. *Nature* 471:336–340.

Chang G, Spencer RH, Lee AT, Barclay MT, Rees DC. 1998. Structure of the MscL homolog from Mycobacterium tuberculosis: a gated mechanosensitive ion channel. *Science* 282:2220–2226.

Chung BC, Zhao J, Gillespie R a, Kwon D-Y, Guan Z, Hong J, Zhou P, Lee S-Y. 2013. Crystal structure of MraY, an essential membrane enzyme for bacterial cell wall synthesis. *Science* 341:1012–1016.

Efremov RG, Sazanov LA. 2011. Structure of the membrane domain of respiratory complex I. *Nature* 476:414–420.

Gourdon P, Liu X-Y, Skjørringe T, Morth JP, Møller LB, Pedersen BP, Nissen P. 2011. Crystal structure of a copper-transporting PIB-type ATPase. *Nature* 475:59–64.

Hattori M, Tanaka Y, Fukai S, Ishitani R, Nureki O. 2007. Crystal structure of the MgtE Mg^2+^ transporter. *Nature* 448:1072–1075.

Housden NG, Wojdyla JA, Korczynska J, Grishkovskaya I, Kirkpatrick N, Brzozowski AM, Kleanthous C. 2010. Directed epitope delivery across the Escherichia coli outer membrane through the porin OmpF. *Proc. Natl. Acad. Sci. U.S.A.* 107:21412–21417.

Ramasamy S, Abrol R, Suloway CJM, Clemons WM. 2013. The glove-like structure of the conserved membrane protein tatc provides insight into signal sequence recognition in twin-arginine translocation. *Structure* 21:777–788.

Tsukazaki T, Mori H, Echizen Y, Ishitani R, Fukai S, Tanaka T, Perederina A, Vassylyev DG, Kohno T, Maturana AD, et al. 2011. Structure and function of a membrane component SecDF that enhances protein export. *Nature* 474:235–238.

Zimmer J, Nam Y, Rapoport TA. 2008. Structure of a complex of the ATPase SecA and the protein-translocation channel. *Nature* 455:936–943.
